# Supplementary material for: MetaRibo-Seq measures translation in microbiomes
Source: Nat Commun. 2020 Jun 29;11:3268. doi: 10.1038/s41467-020-17081-z (PMC7324362; doi:10.1038/s41467-020-17081-z)
Supplement: Supplementary file 10 — Supplementary Data 7 [file 41467_2020_17081_MOESM10_ESM.zip › File2/Confidence_VeryHigh_Taxonomy/200535_out.krona.html]

Javascript must be enabled to view this page.

members
magnitude
magnitudeUnassigned
count
unassigned
taxon
rank

200535\_out

21

21
2
superkingdom

4
1224

SRS019129\_contig\_number\_14212SRS045244\_contig\_number\_contig-100\_10490.10491SRS142781\_contig\_number\_10789SRS142890\_contig\_number\_contig-100\_25837.128138
21
phylum

17
1236
class

17
91347
order

family

SRS146996\_contig\_number\_9150SRS148159\_contig\_number\_14411
17
2
543

561
1
genus

SRS140492\_contig\_number\_30689
14


SRS012273\_contig\_number\_8752SRS014683\_contig\_number\_contig-100\_1119.1119SRS017191\_contig\_number\_19044SRS017521\_contig\_number\_31602SRS019601\_contig\_number\_12869SRS049402\_contig\_number\_16612SRS076876\_contig\_number\_contig-100\_2320.2321SRS098881\_contig\_number\_16240SRS144219\_contig\_number\_contig-100\_817.818SRS144362\_contig\_number\_42404SRS146888\_contig\_number\_11686SRS147022\_contig\_number\_14663SRS971275\_contig\_number\_contig-100\_15238.159866
13
species
562

1
547
genus

1

SRS1055050\_contig\_number\_29
539813
species
